# Brazilian authorship gender trends on academic surgery: a bigdata analysis

**DOI:** 10.1590/acb397724

**Published:** 2024-11-29

**Authors:** Ana Kim, Luana Baptistele Dornelas, Luiza Telles, Ayla Gerk, Sarah Bueno Motter, Sarah Lopes Salomão, David Mooney, Cristina Camargo, Roseanne Ferreira

**Affiliations:** 1 Centro Universitário São Camilo – School of Medicine – São Paulo (SP) – Brazil.; 2 Faculdade de Ciências Médicas da Santa Casa de São Paulo – School of Medical Sciences – São Paulo (SP) – Brazil.; 3 Instituto de Educação Médica – Rio de Janeiro (RJ) – Brazil.; 4 Harvard University – Medical School – Program in Global Surgery and Social Change – Boston (MA) – United States of America.; 5 Universidade Federal de Ciências da Saúde de Porto Alegre – Medical School – Porto Alegre (RS) – Brazil.; 6 Universidade de São Paulo – Hospital de Anomalias Craniofaciais – Programa de Pós-Graduação em Ciências da Reabilitação – São Paulo (SP) – Brazil.; 7 Boston Children’s Hospital – Boston (MA), United States of America.; 8 Universidade de São Paulo – Faculdade de Medicina – São Paulo (SP) – Brasil.; 9 University Health Network – Division of Urology – Toronto, Canada.

**Keywords:** Gender Equity, Authorship, Education, Medical, Brazil, Developing Countries

## Abstract

**Purpose:**

To evaluate the gender distribution of first and last authors with Brazilian surgical affiliations in PubMed-indexed surgical journals.

**Methods:**

Data from eligible surgical journals were retrieved using Scimago Journal & Country Rank 2021 and manually reviewed. Manuscripts published from 2018 to 2022 were included if at least one author was affiliated with a Brazilian institution and a surgical specialty.

**Results:**

Data from 340 eligible surgical journals were included. We analyzed first and last authors’ forenames of 1,881 manuscripts. Women comprised 16.7% of the first and 12.4% of the last authors. Analyzing the differences in gender trends in authorship across the five Brazilian regions, we found that the South had the highest representation, while the Midwest and North showed the lowest, respectively. Obstetrics and gynecology featured the highest percentage of women-first authors, whereas orthopedics had the lowest. For the last authorship, pediatric surgery showed the highest, with hand surgery having the lowest representation. Male first authors were 1.9 times more likely to engage in international collaborations.

**Conclusions:**

This study suggests the persistent underrepresentation of Brazilian women in surgical journal authorship. Local policy changes should be considered to encourage greater diversity and inclusivity in surgical research.

## Introduction

Brazil has observed an ascending trend in the proportion of women entering the medical profession; as of 2022, women constitute 48.6% of the physician workforce. Despite this, substantial gender disparities persist across various specialties. Data from the Brazilian Medical Demography from 2023^1^ highlight that a pronounced gender gap persists in surgery. Women comprise fewer than 15% of surgeons in urology, orthopedics and traumatology, neurosurgery, cardiovascular surgery, digestive surgery, and thoracic surgery^1^.

This imbalance extends into the surgical academia^2^. Previous studies regarding Brazilian surgical journals have shown that only about 20% of articles had women as first and last authors^3^^,^ 4. Additionally, women rarely reach leadership positions in academia, as illustrated by the “leaky pipeline” theory^5^, with the decline in the numbers of women at each step up the professional ladder^6^. In Brazilian public universities, only 12% of the department chairs within the surgical departments were women^3^^,^ 4. With the frontier of knowledge mainly outlined by men, this can reinforce a cycle as fewer women present papers and gain visibility and recognition.

Considering these findings, it is imperative to analyze the current gender diversity of academic surgery in Brazil. Therefore, we evaluated the publication pattern of Brazilian surgical researchers in worldwide surgical journals. We analyzed the gender distribution of Brazilian first and last authors in surgical journals, their medical specialties, and their distribution across Brazilian regions, international collaborations, and affiliations.

## Methods

### Study design

The study is a cross-sectional bibliometric analysis conducted following an adapted version of the Strengthening the Reporting of Observational Studies in Epidemiology (STROBE) statement to ensure methodological rigor^7^^,^ 8, while using a Big Data methodology and data repository^9^. To address the objectives of our study, we concentrated on a specific aspect within the bibliometric framework.

### Journal selection

Surgical journals were selected using the Scimago’s Journal and Country Rank indexed 2021 list^8^. The eligibility criteria were:

- Active journals indexed on PubMed;
- Journals written in English, Portuguese, or Spanish;
- The journal’s primary scope was surgery.

Manuscript record identification procedure

We utilized PubMed to identify articles published between January 1, 2018 and April 30, 2022 and filtered for eligible journals. A list of indexed articles was retrieved and uploaded into the Entrez Direct (EDirect) software^10^, generating a .txt output file with variables. We extracted data for the following PubMed XML article metadata fields^11^:

- PubMed Unique Identifier (PMID);
- title;
- International Organization for Standardization (ISO) abbreviation journal name^12^;
- Country of the journal;
- Article date of publication;
- Publication type;
- Language;
- First author’s forename;
- First author’s affiliation;
- Last author’s forename;
- Last author’s affiliation;
- Country of the institution.

These variables were chosen to provide comprehensive information about the articles, authors, and journals. The .txt output from EDirect was processed using the Microsoft Excel Power Query^13^ to remove duplicates and extract data on the variables of interest. To access E-Utilities in EDirect^10^, we used the UNIX terminal environment in the Cygwin software version 16.6^14^.

Once the dataset with all variables of interest for all manuscript records was generated, we manually processed the data applying the following inclusion criteria: each article identified must have at least one author affiliated with a Brazilian institution. Records were excluded if they were missing data for the author’s name or affiliation and if the author’s primary affiliation was not from a surgical specialty.

The identification of author affiliation was initially conducted using the Python software, searching the “country of the institution” column with the keywords Brazil or Brasil. In the second stage, two authors manually reviewed the process.

### Variables of interest

The variables of interest for the first and last authors were: gender distribution, geographical distribution, surgical specialty of authors, surgical national distribution using the 2023 National Medical Demographic Census^1^, country of journal (Brazilian vs. international), and, lastly, distribution among the top 10 rated surgical journals.

### Gender classification

Gender definition in this study followed the author’s forename. The first and last authors’ forenames were submitted to a previously validated multinational database (Gender API)^13^ for gender prediction. The software Gender-Api uses publicly available data, governmental data, and manual additions/corrections as data sources to predict someone’s gender based on their forename. It provides the certainty of the assigned gender (in percentage) and the number of data rows examined in the software dataset to calculate the certainty of the assigned gender. Assigned genders with an accuracy of < 80% were manually reviewed by two authors, and discrepancies were decided by consensus.

### Country of affiliation

We relied on the List of ISO 3166-114 to record country names and their variations^12^. Brazilian authors’ geographical regions in Brazil were identified based on their reported primary affiliation.

### Specialty classification

Authors’ specialties were determined based on their reported primary affiliation. Surgical specialties were classified into anesthesiology, breast surgery, general surgery, gastrointestinal surgery, hand surgery, head and neck surgery, neurosurgery, obstetrics and gynecology (GO), oncology surgery, orthopedics/traumatology, pediatric surgery, plastic surgery, cardiothoracic surgery, vascular surgery, and urology. The specialty classification was done manually by two authors.

### Journal classification

The 2021 Scimago Journal & Country Rank^8^ indicator was utilized to analyze gender distribution within the 10 highest-ranked surgical journals. We used this information as a proxy for journal visibility.

Using Excel Microsoft Excel, we assessed the country of the journal for interpretation.

### Statistical analysis

Descriptive statistics involving frequencies and percentages were employed to summarize the distribution of citations. All variables collected were categorical. χ^2^ tests were used for inferential analysis to examine differences in the percentage of women authors across years, regions, specialties, and authorship roles. When multiple pairwise comparisons were applicable, significance was adjusted using the Bonferroni’s post-hoc test. A logistic regression analysis was conducted to determine the factors predicting women’s first authorship. A similar analysis was performed to evaluate predictors of gender of last authorship. Odds ratios, adjusted odds ratios, and 95% confidence intervals were reported for each independent variable. A *p* < 0.05 was considered statistically significant. Statistical analyses were performed using Stata 18 BE^15^.

## Results

### Overview of included studies

**Figure 1:**
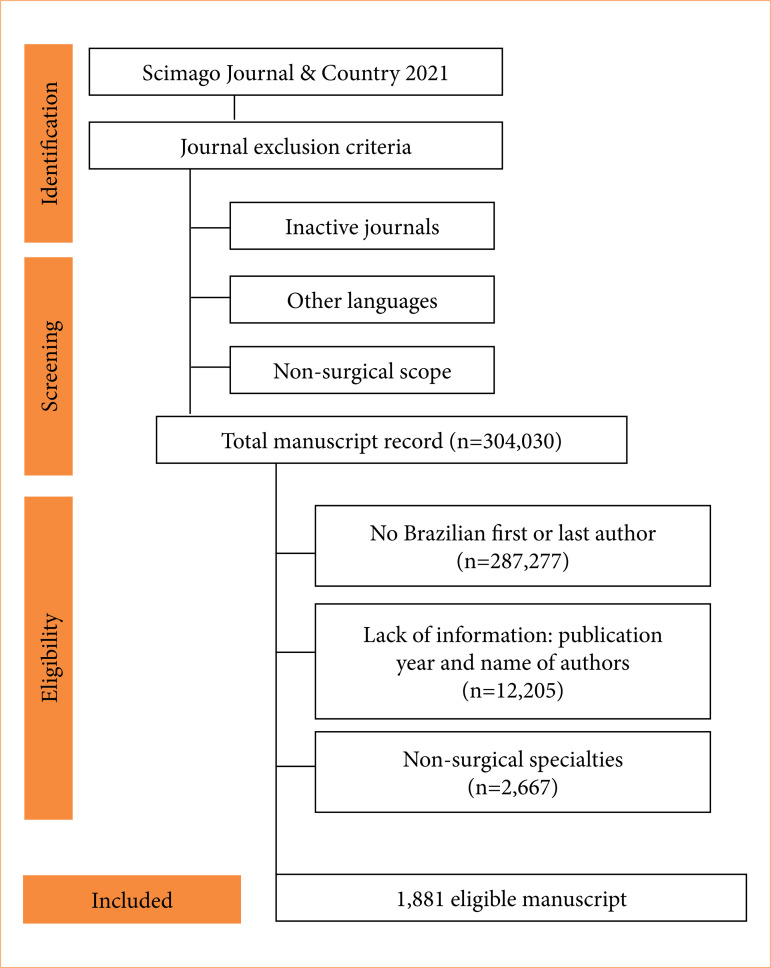
Manuscript selection flowchart.

Source: Elaborated by the authors.

Of the 492 surgical journals searched, 340 met the inclusion criteria. We retrieved 304,030 manuscript records. Among them, 287,277 records were excluded because neither the first nor the last author were affiliated with a Brazilian institution. We further excluded 12,205 records that lacked a publication year or the first or last authors’ names. Of all, 262 citations, wherein gender accuracy for the forename was below 80% as per the Gender-API, were reviewed by two authors; 32 of these observations were amended. We excluded manuscripts when the first authors were dentists: 633; basic scientists: 139; non-healthcare professionals: 335; non-physician healthcare professionals: 289; non-surgical specialists: 301; and unknown surgical specialty: 612. Manuscripts were excluded when last authors were dentists: four; basic scientists: 26; non-health care professionals: 78; non-physician healthcare professionals: 38; non-surgical specialists: 84; and unknown surgical specialty: 130. Ultimately, we analyzed 1,881 eligible manuscripts (Fig. 1).

### Overall results

**Figure 2:**
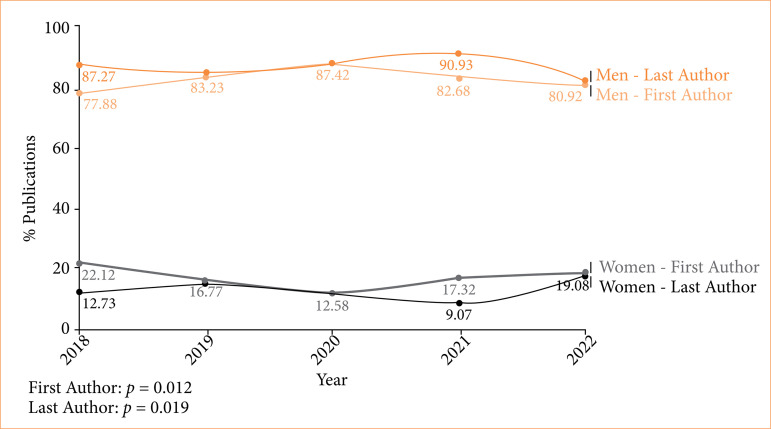
Gender representation on publications between 2018 and 2022.

Source: Elaborated by the authors.

Women constituted 16.7% (314/1,881) of first authors and 12.4% (234/1,881) of last authors (Table 1). Over the five years, the proportion of publications by female first authors remained relatively constant. In 2019, women made up 16.8% (52/310) of first authors, and this figure was 17.3% (84/485) in 2021. The same happened with the representation of female last authors over this period. The proportion of publications by female last authors was from 15.2% (47/310) in 2019 to a slight decrease of 9.1% (44/485) in 2021 (*p* < 0.001) (Fig. 2).

### Gender distribution from medical specialties

**Figure 3:**
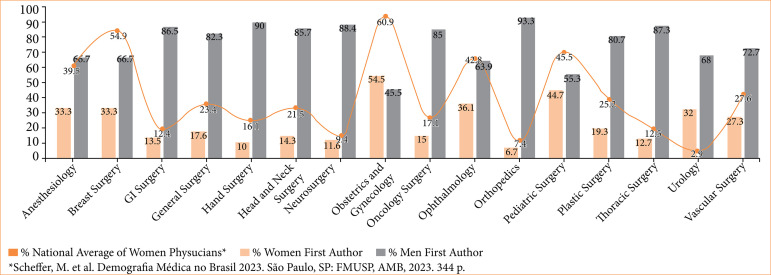
Gender distribution by specialty for first authors.

Source: Elaborated by the authors.

**Figure 4:**
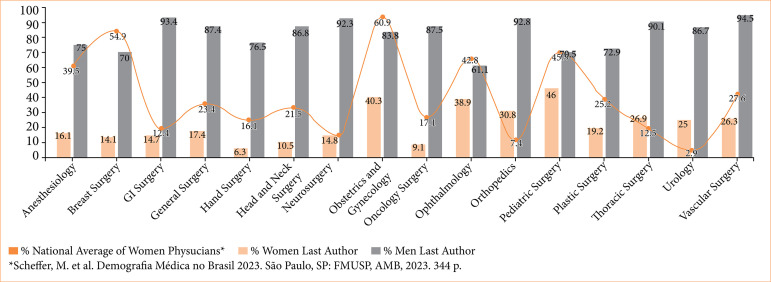
Gender distribution by specialty for last authors.

Source: Elaborated by the authors.

Women’s representation varied significantly across subspecialties (*p* < 0.001). Obstetrics and GO featured the highest proportion of female first authors (54.5%, 18/33), whereas orthopedics had the lowest (6.7%, 16/238) (Fig. 3). For the last authorship, pediatric surgery showed the highest representation of women (46%, 13/44), with hand surgery having the lowest (6.3%, 4/18) (Fig. 4). A table with the total number of publications by gender can be found in Table 1.

### Geographical regions

**Figure 5:**
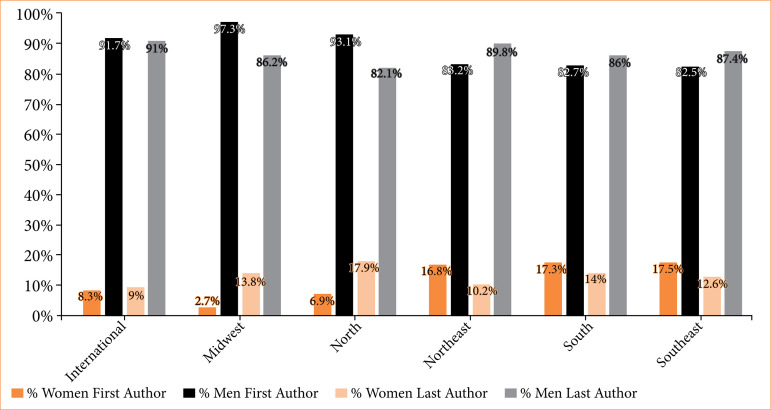
Geographical distribution of authorship.

Source: Elaborated by the authors.

Representation of first and last authors varied significantly across geographical regions in Brazil (*p* < 0.001). Although 73.2% (224/306) of all female first authors’ publications originated from the Southeast region, institutions in the South boasted similar high representation of women in first (17.3%, 49/283) and highest for last (14%, 34/243) author positions. Conversely, institutions in the Midwest (2.7%, 1/37 for first authors) and Northeast (10.2%, 13/128 for last authors) displayed the most pronounced underrepresentation of women in these authorship positions (Fig. 5).

### National and international collaborations

In total, 80.7% (1,519/1,881) of authors collaborated with peers from their own geographical regions. Female first authors were more likely to collaborate with authors from the same region compared to their male counterparts (86.6%, 272/314 for women vs. 79.6%, 1,247/1,567 for men, *p* < 0.001). Last authors had similar rates of national collaboration (82.5% 193/234 for women and 80.5% 1,326/1,647 for men, *p* = 0.535) (Table 1).

Female authors were underrepresented in international collaborations. Male first authors were 1.9 times more likely to collaborate with international last authors than female first authors (odds ratio–OR = 1.9, 95% confidence interval–95%CI 1.2–3.0). The OD was lower for male last authors (OR = 1.4, 95%CI 0.6–3.1) collaborating with a first author from a different country. Only 7% (22/313) of female first authors participated in collaborations with international last authors, compared to 12.6% (197/1,559) of male first authors (p = 0.004). In contrast, rates of international collaboration for last authors were the same (3% (7/230) for female last authors vs. 4.2% (69/1,636) for male ones, *p* = 0.479).

### Journal distribution

Of all the 1,881 citations included, 19.9% (375/1,881) were published in Brazilian journals. From these 375 publications, 83.7% featured male first authors, while only 16.3% had female first authors. When examining last authors in national journals, 90.7% were men and 9.3% were women (Table 1).

**Table 1:** Characteristics of gender distribution.

Characteristics	n	%
Total number of surgical journals included	340	100
First or/and last author affiliated with a Brazilian institution manuscript	1,881	100
**First author**		
Men	1,567	83.3
Women	314	16.7
**Last author**		
Men	1,647	87.6
Women	234	12.4
**First and last author collaboration**		
Female first author – Female last author	83	26.4
Female first author – Male last author	231	73.6
Male first author – Female last author	151	9.6
Male first author – Male last author	1,416	90.4
**Journal type**		
Female first author national journal	61	16.3
Female first author international journal	253	16.8
Male first author national journal	314	83.7
Male first author international journal	1,253	83.2

Source: Elaborated by the authors.

### Brazilian authorship among the top 10 journals

Three of the top 10 journals (*European Urology Oncology, Journal of Bone & Joint Surgery,* and *American Journal of Surgical Pathology*) analyzed did not feature any contributions from authors affiliated with Brazilian institutions. Sixteen first authors and 16 last authors published in the top 10 journals. Only 14.3% (2/16) of the first or last authors were women.

**Figure 6:**
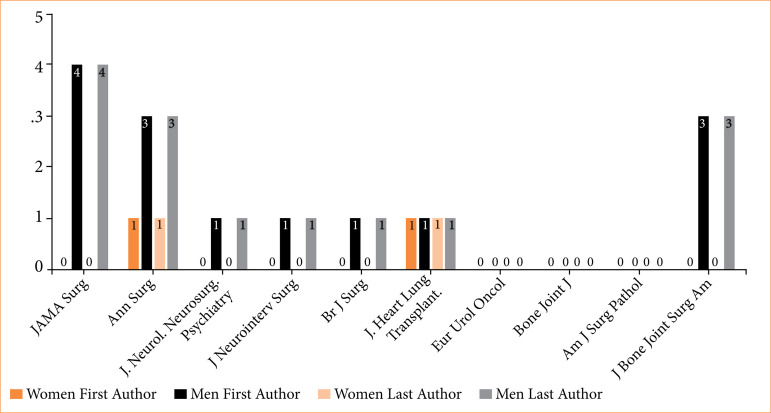
Authorship distribution on top 10 surgical journals.

Source: Elaborated by the authors.

Additionally, five journals (*JAMA Surgery, Journal of Neurology, Neurosurgery and Psychiatry, Journal of Neurointerventional Surgery, British Journal of Surgery*, and *Journal of Bone & Joint Surgery*) had no women participating as first or last author, yet they had at least one male Brazilian author in these positions (Fig. 6).

## Discussion

The present study highlights the disparity in Brazilian gender authorship in articles published in surgical medical journals. Similar studies analyzed the level of female surgical authorship in Brazilian journals^3^^,^ 4. While the presence of women in medicine and surgery in Brazil has exhibited a steady rise, an unsettling underrepresentation of female authorship persists, particularly in surgical specialties^3^. In this study’s analysis of surgical journals featuring Brazilian authors worldwide in the last four years, women accounted for 16.7% of first authors and 12.4% of last authors.

These findings align with existing literature. For instance, Graner et al.^4^ identified a persistent gender inequity in authorship in Brazilian surgical journals. They also report stable inequitable rates of female as first and last authors from 2015 and 2019. Interestingly, our results reported similar stability, adding concern to the slow progress made over time. Moreover, we observed that the proportion of female first authors slightly decreased in 2020 and 2021, and female last authors rates also decreased in 2020, yet subtly increasing in 2021.

One possible explanation for the decline in first and last author’s authorship rates in 2020 is the impact of the COVID-19 pandemic. During this time, many female researchers had added challenges in balancing their multiple roles simultaneously, including domestic duties, parenting, children’s education, and various other responsibilities^16^^,^ 17. This has been documented throughout the pandemic, with a reported decrease in submissions, new projects, and publications among female researchers, particularly those who were mothers^18^^,^ 19. This issue, however, extends beyond the pandemic, with physicians who are mothers bearing a disproportionate burden of childcare and household responsibilities^20^, with consequential increased rates of depressive and anxiety symptoms^21^.

While this is no novelty, the pandemic likely intensified the demands, increasing the impact on their personal and professional lives. A study involving female faculty at an urban medical education center revealed that, compared to their male counterpart, women were more likely to refuse leadership opportunities before and during the pandemic due to their responsibilities for childcare and household tasks^20^. Moreover, although manuscript submissions increased in 2020, male authorship remained higher than female authorship, especially among junior faculty members^21^ and in health and medicine journals^19^.

### Gender disparities in surgical specialties

The disparities in authorship can be even more alarming across different surgical specialties. The highest proportion of female first authors was observed in obstetrics and GO (54.5%, 18/33), whereas the lowest proportion was seen in orthopedics (6.7%, 16/238).

When considering last authorship roles, pediatric surgery (46%, 13/44) displayed the highest representation of women, while hand surgery (6.6%, 4/18) the lowest. This is similar to studies conducted in high-income countries, which have consistently demonstrated the same pattern of gender disparity in academic publications in surgical subspecialties, such as obstetrics and GO (71% female first authors)^22^, orthopedics (15.4% female first authors)^23^, pediatric surgery (26.4% female last authors)^24^ and hand surgery (14.2% female last authors)^25^.

While obstetrics and GO exhibited the highest representation of women as first authors and pediatric surgery demonstrated the highest proportion of women as last authors, these figures remain below the 55% mark, underscoring a clear and persistent disparity between the percentage of women in these specialties and female authorship. This supports the inference that the number of female-led publications is not directly proportional to the number of women in each specialty. This phenomenon becomes evident when conducting a comparative analysis of gender proportions in surgical specialties in our study and the Brazilian national distribution based on medical demographic data^1^.

A higher proportion of women in a field was not necessarily correlated with research productivity. In obstetrics and GO, 60.9% of surgeons are women, and 54.5% of publications had women as the first author. In contrast, urology has only 2.5% women^1^, but, surprisingly, urology does not have the lowest proportion of female authors to male authors, with 32% of first authors women. Regarding the last author, in pediatrics, the proportion of women compared to the national distribution^1^ (45%) was similar to the proportion of female last authors (46%, 13/44). On the other hand, hand surgery, despite a national distribution of women of 16.1%, only 6.6% (4/18) of publications had women as the last author^1^.

Many modifiable institutional and cultural factors may contribute to these demonstrated gender disparities^26^. For instance, women often encounter fewer research opportunities in their early careers. To illustrate this scenario, Lopez et al.^27^ identified a gender disparity at the outset of first authorship that decreased subsequently in the last author in ophthalmology. Moreover, one of the significant factors contributing to low research among female first authors is pregnancy^28^. Studies have shown that pregnancy can be a hindrance to academic production^29^ and having more children decreases productivity. Having one child is associated with a loss of 9.5% of productivity, 22% with two children, and 33% with three children^30^. This does not happen with male authors, as they do not experience any significant variability in productivity with parenthood^30^.

Another contributing factor could be the lack of female role models to emulate, particularly in high-ranking positions, which aligns with the leaky pipeline theory. The scarcity of women in senior roles, including university positions, is striking. A meta-analysis conducted on full professorship among academic physicians revealed that men had 2.77 times higher odds of attaining full professorship than women^31^. Notably, the specialty with the most significant gender difference in full professorship was obstetrics and GO^31^^,^ 32, despite having one of the highest proportions of female professionals.

### Women’s regional representativeness

Brazil is a vast and diverse country, with various healthcare and educational resources spread across different regions^33^. The Southeast region, containing Sao Paulo and its prestigious universities and medical centers, is rich in resources^33^. The Midwest and North regions are not^33^. Our findings revealed that, while most female-authored publications were from the Southeast region, the proportion of female first authors remained below 30% (28.31%), nearly identical to the North (28.85%). Regional disparities appeared when considering the first and last authors’ positions combined, similar to previous studies evaluating low- and middle-income authorship trends^34^. This resonates with the intricacies of our national cultural and historical context^33^ and can be insightful to other LMICs and countries with similar characteristics^33^^,^ 35 ^–^ 37. These can foster policymaking to improve professional environments and sustain academic development for women across various resource settings.

### International contribution

As we explore the regional disparities in authorship representation, it is worth examining how international collaboration patterns differed among first and last authors. Recent studies examining gender disparities in international research collaboration have revealed an interesting trend: male first and last authors were approximately two times more inclined to engage in international collaborations compared to their female counterparts^39^^,^ 40. Even though these researchers explored various fields, their contributions have contributed to our understanding. Our study aligns with these findings, as we observed a similar trend: female first authors collaborated internationally less than male authors, with only 7% of female first authors engaging in international collaboration (*vs*. 12.6% male first authors, *p* = 0.004) and no difference between last authors (3% for female *vs*. 4.2% for male, *p* = 0.399).

Gender disparities in authorship were more significant when we analyzed high-impact journals, whether originating in Brazil or globally. In a recent study examining the gender distribution among first and last authors in the 10 top surgical journals from 2015 to 2022, only 22.3% of first authors were female, as were 14.3% of the last authors^38^. In our study, the rates were similar, with 22% of first authors and 7.9% of last authors female. Notably, three of the top 10 journals had no female first or last authors: *JAMA Surgery, Journal of Neurointerventional Surgery* and *British Journal of Surgery* 32 ^,^ 39 ^–^ 41. Moreover, there were no Brazilian authors in two journals: *Journal of Bone & Joint Surgery* and *American Journal of Surgical Pathology*.

### Surgical culture

The challenge faced by women in academic surgery is multifaceted and largely stems from largely male-oriented social norms that create a sense of belonging uncertainty for them and underrepresented or marginalized minorities^42^. These environments also fuel gender bias, which can manifest in various forms, from discrimination to microaggressions and macroaggressions that negatively impact women’s careers^42^.

Disparities persist in terms of compensation, with female surgeons earning 8% less annually^43^, even after controlling for factors such as training, subspecialty, faculty rank, and clinical and academic productivity metrics. Additionally, the challenging balance of professional and home responsibilities remains an unspoken challenge for many women in academic surgery, particularly in dual-professional families, exacerbating the so-called “double burden” of unpaid household work^44^. These issues underscore the importance of addressing both gender inequalities and the social norms that perpetuate these disparities in order to promote a more inclusive and equitable academic surgery field.

As evidenced, worldwide women’s presence in academic surgery authorship, notably leadership positions, remains inadequate^45^. However, some countries have developed strategies to diminish these gaps. Over 30 years ago, the John Hopkins University School of Medicine established an Equity Issues Task Force from 1990 to 1995 to identify barriers and enhance representation for women, resulting in an increase in 550% of women-associated professors^46^. These actions emphasize the importance of fostering national and regional strategies to promote further career advancement, professional development, and research opportunities for women in surgical, anesthesia, and obstetrics specialties. Moreover, implementing mentorship and guaranteeing representation is a must^45^ and crucial in narrowing gender gaps, fostering an inclusive environment, and promoting equitable growth for female surgeons.

Sex and Gender Equity in Research (SAGER) Guidelines^47^ are deemed effective and highly endorsed by academic stakeholders and should be implemented by journal editors. These policies will help journal editors to contribute to standardizing terminology and will support best reporting practices for inclusion of sex and gender as variables. In addition, journals should adopt a clear Equal Employment Opportunity and Diversity Statement for implementing inclusive environments^48^. A Brazilian study examining four academic journals revealed a stark gender disparity in their scientific committees. Only three of 158 scientific committee members on two journals were women, representing a mere 1.9% of the composition. Notably, the journal *Revista do Colégio Brasileiro de Cirurgiões* stood out as an exception, with women comprising 93% (67/72) of its reviewers. In contrast, one particular journal, *Revista Brasileira de Cirurgia Cardiovascular*, exhibited a complete absence of female participation, both in its scientific committees and among its reviewers^49^.

### Limitations

This study is not without limitations. First, the author’s gender was obtained using the author’s first name and analyzed by Gender-API software, which works with limited binary gender (woman or man). Second, the data was analyzed manually. Therefore, there are limitations in precision compared to data analyzed by software. Third, affiliation information has been provided by the authors, with some indicating more than two different specialties. Fourth, our findings explored a limited number of journals. Lastly, the validation of the adapted version of the STROBE Statement. Despite these weaknesses, this study evaluated bibliometric literature using a systematic manner and an accurate gender software assessor. We highlighted disparities across regions and specialties that have contributed to equity in authorship in surgery.

## Conclusion

Despite an increase in the proportion of women in surgery over the past years, women remain underrepresented as first and last authors in high-impact journals and international collaborations, even when working in high-resource regions. Future research should explore the factors limiting women’s engagement in surgical research and assess potential strategies to enhance their leadership in surgical publications.

## Data Availability

The data that support the findings of this study are available from the corresponding author, upon reasonable request.
